# Enhancing insulin administration safety in inpatient care: findings from a root cause analysis and continuous improvement intervention

**DOI:** 10.1515/med-2026-1383

**Published:** 2026-06-05

**Authors:** Qing Wang, Yanan Ma, Min Li, Jinyu Song, Hongli Ma

**Affiliations:** Department of Otolaryngology – Head and Neck Surgery, Shanxi Bethune Hospital, Shanxi Academy of Medical Sciences, Third Hospital of Shanxi Medical University, Tongji Shanxi Hospital, Taiyuan, China

**Keywords:** medication errors, nursing adverse event, nursing practice, patient safety, risk management, root cause analysis

## Abstract

**Objectives:**

This study aimed to quantify the incidence of insulin-related medication errors among hospitalized patients, identify underlying causal factors through root cause analysis (RCA), and evaluate the effectiveness of continuous improvement strategies in mitigating nursing adverse events associated with insulin administration.

**Methods:**

A 25-week prospective quasi-experimental clinical study was conducted at a tertiary care hospital in Shanxi Province. Patients were assigned to either a control group receiving standard management or an intervention group managed under a continuous improvement protocol. Group assignment was determined by the chronological order of hospital admission, with the initiation of the intervention serving as the demarcation point. Primary outcomes included the frequency of insulin-related medication errors, adherence to established safety check protocols, and changes in nursing staff safety practices and psychological resilience.

**Results:**

RCA identified key contributing factors to insulin-related medication errors, including the absence of a comprehensive management framework, inadequate staff training, limited pharmacological knowledge among nursing personnel, and insufficient diversity in patient education strategies. A total of 123 patients were enrolled, with 65 in the intervention group and 58 in the control group. The intervention group demonstrated a significantly lower incidence of insulin-related medication errors compared to the control group (p<0.001). Additionally, patients in the intervention group exhibited improved glycemic control, and nursing staff showed enhanced adherence to safety protocols and greater psychological resilience (p<0.001).

**Conclusions:**

Conducting an RCA enabled the identification of critical factors contributing to insulin-related medication errors and informed the development of strategies to reduce these risks. The results underscore the importance of implementing continuous improvement approaches to enhance patient safety. These findings provide actionable recommendations for improving nursing practices and healthcare management to optimize safety outcomes.

## Introduction

Diabetes mellitus is a chronic, highly prevalent condition that has emerged as a major global public health concern in recent years. According to a 2022 statistical bulletin issued by the Chinese government, the prevalence of diabetes in China has reached 11.9 %, affecting approximately 125 million individuals [1], [2], [3]. Insulin and its analogues play a key role in the management of diabetes, just like prevention through therapeutic education, insulin clinical use is associated with significant challenges.

Many insulin formulations possess similar trade names, packaging designs, and dosage forms, which increases the potential for confusion and elevates the risk of medication errors relative to other properties.

Medication errors, defined as omissions or inaccuracies in the administration or management of medications, can lead to temporary or permanent patient harm and, in some cases, pose life-threatening risks [4]. These incidents undermine the foundational principles of patient safety within healthcare systems and may also result in reputational damage to healthcare institutions. Furthermore, such incidents can cause psychological or emotional distress, referred to as “secondary harm,” to affected patients.

An adverse event (AE) is defined as an “event resulting in accidental injury to a patient.” Medication errors are considered a common subtype of AEs and occur frequently in nursing and broader healthcare settings. These events may result in physical harm and require immediate assessment by nursing staff to determine their severity, as well as consultation with physicians to guide clinical decision-making and avoid unnecessary or inappropriate treatment. Nursing staff play a critical role throughout the medication-use process. According to the study, among the safety-related processes and steps during medication administration, the incidence of AEs is highest at the drug administration phase [5].

The healthcare environment significantly influences the occurrence of unsafe behaviors. Hospital settings are complex systems involving both physical elements (e.g., medical devices, equipment, and consumables) and human factors (e.g., clinical competencies, staffing levels, and resource availability). Among these, human factors are especially critical in preventing AEs. The systematic analysis of AE data, particularly those involving medication errors, is essential for risk identification and safety assurance in clinical nursing practice. Effective risk management is particularly important in nursing workflows, including the use of nursing materials and the allocation of human resources.

Accurate identification of the causes of AEs and the development of strategies to prevent recurrence require the application of rigorous analytical methods. Root cause analysis (RCA), originally developed in the United States, is a retrospective method used for investigating and understanding error causation. RCA can be categorized into two forms: individual RCA and integrated RCA. Individual RCA focuses on a single AE with serious outcomes, such as permanent loss of function or mortality due to a critical medication error. In contrast, integrated RCA addresses systemic issues by analyzing a series of similar AEs that occur within a defined time frame. This approach is retrospective, systematic, and preventive, and it facilitates the identification of latent errors and contributing factors, thereby supporting the development of targeted interventions to prevent recurrence. RCA has demonstrated substantial utility in nursing risk management and has produced favorable outcomes [6].

To date, due to the limitations of specialized nursing resources, no studies have employed an integrated Root Cause Analysis (RCA) approach to analyze insulin-related medication errors or evaluate the clinical effectiveness of corresponding intervention strategies. Al Mardawi et al. [7] explored the application of Root Cause Analysis and Action (RCA^2^) in medication safety incidents, emphasizing its role in identifying system failures and human factors, and noted that abbreviated RCA^2^ can reduce harm from non-sentinel events. Zhang et al. [8] applied RCA to the management of nursing medication errors, and through systematic analysis, reduced the incidence of medication errors and improved patient satisfaction. However, these studies did not involve a specialized analysis of insulin-related medication errors. He et al. [9] conducted a root cause analysis of insulin medication errors based on TeamSTEPPS, and prevented the recurrence of similar events through modular training and process optimization. Nevertheless, their research focused on case analysis and did not involve a systematic exploration of integrated RCA for a series of insulin-related AEs. Hidalgo-Velasco et al. [10] conducted a study in a Mexican pediatric hospital and used RCA to identify risk factors (such as work overload) associated with medication AEs, but did not involve insulin administration scenarios and did not adopt an integrated RCA method. These studies have largely overlooked the application of integrated RCA in the analysis of insulin-related medication errors and the evaluation of intervention effects.

Therefore, this study aims to achieve the following objectives: (1) Identify the root causes of insulin-related medication management errors involving nursing staff through RCA; (2) Develop targeted improvement strategies based on the identified factors; (3) Evaluate the clinical effectiveness of these strategies in optimizing the prevention of insulin administration errors.

## Data and method

### Patient enrollment

The formula for sample size calculation was applied as n_1_=n_2_=[Z_1−_α/_2_√(2p̄(1_−_p̄)) + Z_1−_β√(p_0_(1_−_p_0_) + p_1_(1_−_p_1_))]^2^/(p_0_ − p_1_)^2^ First, the “incidence of insulin medication errors” in our hospital from 2022 to 2023 was reviewed, which was approximately 40 %. Combined with clinical practical expectations, the incidence of medication errors in the intervention group was set to be reduced to less than 10 % to confirm the clinical significance of the intervention. Therefore, the expected intervention effect size was determined as 30 % (40–10 %). Next, the significance level was set at α=0.05 (two-tailed test) to control the incidence of Type I error (false positive) within 5 %. The statistical power was set at 1 − β=0.80 (β=0.20) to control the incidence of Type II error (false negative) within 20 %. Additionally, considering potential sample loss among inpatients due to early discharge, transfer to other departments, or incomplete data records, a 10 % sample size increment was added with reference to the loss rate reported in similar studies on inpatients, to ensure an adequate number of valid samples. By substituting the above parameters into the formula, the minimum theoretical sample size for each group was calculated to be 48 cases. Accounting for the 10 % sample loss rate (48 × 10 %≈5 cases), an additional 5 cases were supplemented for each group. Finally, it was determined that the control group and the intervention group should include at least 53 cases each.

In this 25-week prospective quasi-experimental clinical trial, a total of 123 patients diagnosed with diabetes mellitus and admitted to a tertiary hospital in Shanxi Province between June 2023 and June 2024 were enrolled using a convenience sampling method. Of these, 58 patients who received care between June 2023 and December 2023 were assigned to the control group, while 65 patients treated from January 2024 to June 2024 were assigned to the intervention group.

The inclusion criteria were as follows: (1) age 18 years or older; (2) confirmed diagnosis of diabetes mellitus based on clinical assessment and laboratory findings; (3) treatment with any subcutaneous insulin formulation, including rapid-acting, intermediate-acting, long-acting, or premixed types; (4) absence of coexisting endocrine disorders; and (5) inpatient status at the time of study enrollment. The exclusion criteria were as follows: (1) presence of severe cardiovascular, cerebrovascular, renal, or other major organ dysfunction; (2) current or previous diagnosis of psychiatric disorders or severe communication impairments; and (3) incomplete data records.

Written informed consent was obtained from all participants prior to enrollment. The study protocol was reviewed and approved by the Hospital Ethics Committee (Approval No.: YXLL-2023-033).

### Research method

**Figure 1:**
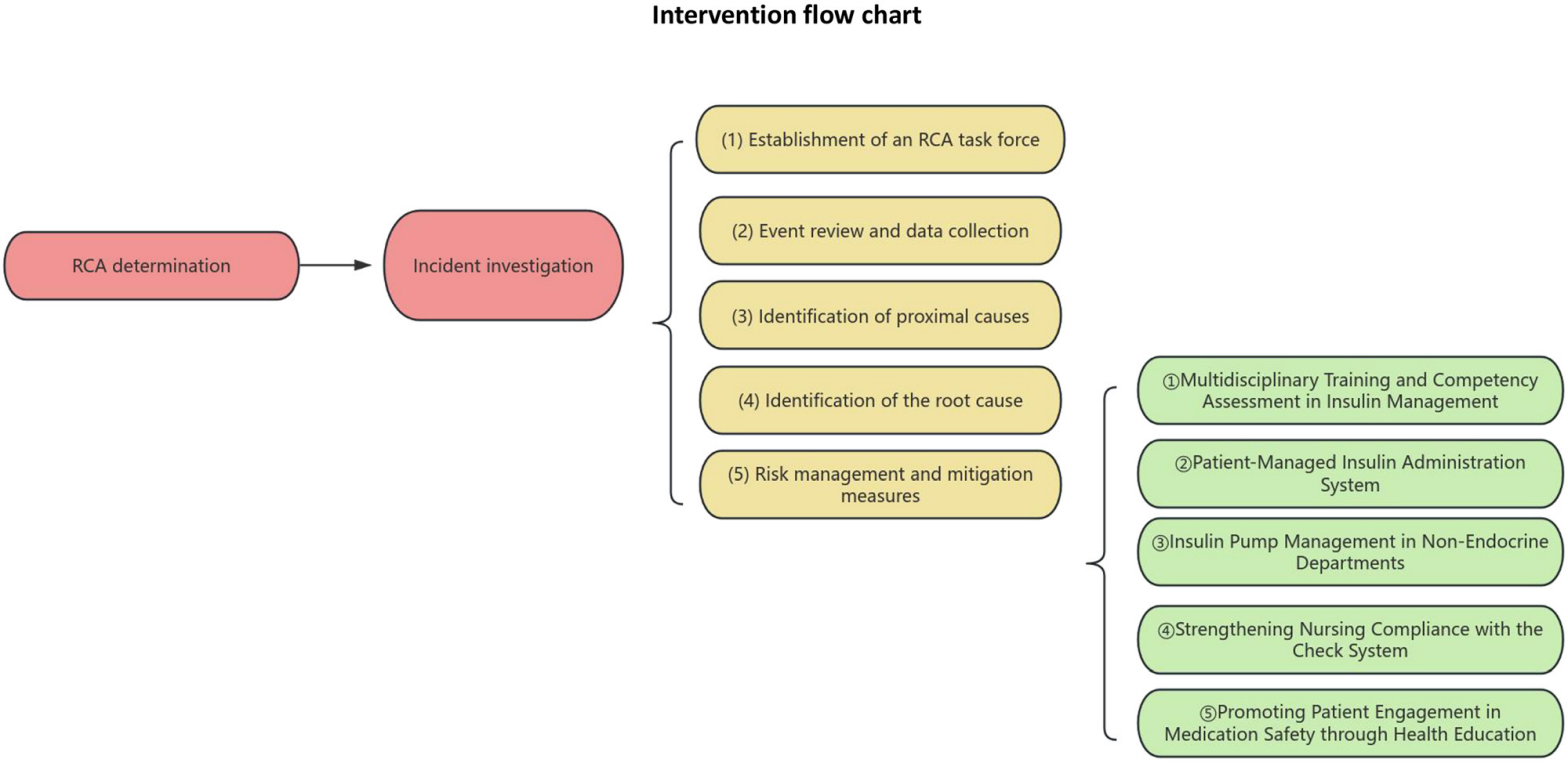
Intervention flowchart.

This quasi-experimental study employed a multi-level data collection approach, incorporating RCA to systematically examine a series of AEs related to medication errors. An integrated RCA method was utilized to identify contributing factors and to develop evidence-based improvement strategies. These strategies were formulated by an interdisciplinary team consisting of nursing staff, physicians, and equipment specialists. Group assignment was determined based on the implementation timeline of the improvement strategy. Patients in the control group received routine nursing care and were managed under standard nursing quality protocols. Beginning in January 2024, the intervention group received care under a revised management approach informed by integrated RCA findings. The specific methods of implementation are described as follows and presented in Figure 1.

#### RCA determination

During routine nursing workflows and standard nursing quality management, a total of 26 AEs related to insulin administration were documented. These included 11 cases of incorrect medication type, 7 cases of inaccurate injection dosing, and 8 cases of delayed insulin administration. These events were associated with abnormal blood glucose fluctuations, increased risks to patient safety, and elevated medical costs for the affected patients. The cluster of AEs was identified as indicative of a systemic issue using an abnormal event decision tree. Based on established severity and risk assessment criteria, the events were classified as level 3 in terms of clinical impact. In response, departmental corrective actions were initiated, and an integrated RCA was conducted to systematically identify and address the underlying contributing factors.

#### Incident investigation

##### Establishment of an RCA task force

To support the RCA process, a dedicated task force was formed comprising two subgroups: an expert panel and an incident-related investigation team, with a total of nine members. The expert panel consisted of three nursing specialists and one equipment expert. Nursing specialists were selected based on their extensive clinical experience and academic contributions in the field, each holding at least a bachelor’s degree and more than 10 years of clinical practice, ensuring high professional standards. The equipment expert was responsible for identifying potential AEs related to the use of insulin delivery devices. The incident-related team included five clinicians and nursing staff members who were experienced in RCA methodology and served as the primary implementation unit. All team members completed structured RCA training and had relevant practical experience. Their familiarity with clinical workflows enabled them to independently conduct investigations and perform targeted causal analyses.

##### Event review and data collection

The task force was further divided into three functional subgroups: the interview group, the equipment group, and the documentation group. The interview group consisted of nursing staff and clinicians. For any AE either directly reported by the responsible nurse or identified by the task force members, the interview group first retrieved electronic medical records to determine whether it constituted a medication-related AE. Subsequently, structured interviews (30 min each) were conducted with direct witnesses, including patients, their family members, and the nursing staff involved in the incident, within 24 h. Group members collected contextual information, documented examples of nursing risk behaviors, and assessed the frequency of similar events among staff. The equipment group inspected the medical devices used during the event and evaluated environmental factors within the ward to identify potential contributing conditions. All medical equipment involved in the investigation was hospital-issued. The documentation group recorded all findings and compiled a detailed chronological timeline of the incident.

##### Identification of proximal causes

To determine the proximal causes of the AEs, first, the research team classified and identified the types of medication errors. For example, wrong insulin type: a discrepancy between the insulin formulation documented in the physician’s prescription and the one actually administered (e.g., incorrectly using rapid-acting insulin aspart instead of long-acting insulin glulisine), which must be confirmed by cross-verifying the electronic information system and nursing records. Wrong dose: Administration of an insulin dose deviating by >10 % from the prescribed dose (e.g., 9 units administered for a 10-unit order, or 11 units for a 10-unit order), with dose calculation verified by two independent researchers. For dose errors involving clinical impact, corresponding blood glucose changes (e.g., fasting blood glucose <3.9 mmol/L for excessive dose, >13.9 mmol/L for insufficient dose) were used as auxiliary validation.

Delayed injection: Insulin administration occurring >30 min after the scheduled time (scheduled times were defined per hospital protocol: rapid-acting insulin 15 min pre-meal, intermediate-acting/long-acting insulin at fixed times 08:00 and 20:00), excluding delays due to patient-related factors (e.g., refusal, temporary medical procedures) documented in nursing records. The team employed a fishbone (Ishikawa) diagram, categorizing potential contributing factors into four domains: personnel, environment, equipment, and methods. These causes were analyzed using trend indicators derived from observational and interview data. The identified proximal causes were then organized into a structured framework, as presented in Table 1.

**Table 1:** Proximal causes of adverse events related to insulin medication errors in patients with diabetes.

Type of event	Proximal causes
Failure of on-time insulin injection	① Incomplete handover of insulin injections ② Insufficient supervision mechanism for self-owned insulin by patients ③ Inefficient patient education on insulin use ④ Inadequate training of nursing staff on hypoglycemia knowledge
Insulin type error	① Inadequate inspection and verification by nurses ② Failure to address incorrect orders from the doctor ③ Lack of warning signs for insulin pump use ④ Absence of management system for patient self-owned insulin
Insulin injection dose error	① Inadequate insulin pump management ② Poor implementation of the nurse check system ③ Lack of patient involvement in safety management

##### Identification of the root cause

The RCA team employed the 5 Whys method [11] to conduct logical analysis, tracing each AE from its initial manifestation and identified proximal causes to its underlying root causes. This structured approach enabled the team to explore multiple layers of causation.

To determine whether a given factor qualified as a root cause, the team applied the following three evaluative criteria:

1) If the cause did not exist, would the problem still occur?
2) If the cause were corrected or eliminated, could the problem recur due to the same cause?
3) If the cause were corrected or eliminated, could a similar situation arise again?

A factor was classified as a root cause only if the answer to all three questions was “no.” Using this method, eight preliminary root causes were identified and subsequently consolidated into five primary root causes based on thematic similarity and causal overlap. The final categorization is presented in Table 2.

**Table 2:** Analysis of contributing factors to adverse events related to insulin medication errors in patients with diabetes.

Root causes	Root causes after integration
1. Inadequate training provided by the department on hypoglycemia	1. Insufficient training on physiological knowledge related to blood glucose and insulin medication precautions by the department.
2. Inadequate training provided by the department on insulin-related knowledge	
3. There is no self-owned insulin management system	2. The hospital has not developed a self-owned insulin management system.
4. The hospital has not formulated insulin pump management system for non-endocrine departments	3. The hospital has not developed an insulin pump management system for non-endocrine departments.
5. Patients with insulin pumps have no conspicuous notice	
6. Nurses don’t understand the meaning of checking	4. The checking system is not effectively implemented due to the lack of understanding by nurses.
7. Education of insulin injection for patients is mainly oral, with education being limited to a single format	5. Training and education on insulin use for patients is limited, lacking comprehensive content. Patients are not encouraged to actively participate in their own safety management.
8. The department does not formulate measures to involve patients in patient safety management	

##### Risk management and mitigation measures

A set of targeted risk management strategies was developed based on integrated RCA findings to address contributing factors to insulin-related medication errors. These strategies focused on enhancing nursing competencies, standardizing insulin use protocols and promoting patient involvement in safety management. The following interventions were implemented:

(1) Multidisciplinary training and competency assessment in insulin management

To improve nursing staff capacity in the prevention and management of AEs, a structured management system was developed encompassing training, assessment, and supervision.

Systematic training: Comprehensive training programs were introduced to reduce insulin-related medication errors and increase awareness of AEs. Data from historical medication errors were reviewed to develop standardized instructional materials. The nursing department collaborated with endocrinologists to design and deliver a series of continuing education courses. Monthly AE analysis meetings and scenario-based simulation training sessions were also conducted to reinforce learning and encourage experiential review.

Optimized assessment: Evaluation methods were refined to include both centralized simulation-based assessments and on-site clinical evaluations. Simulation assessments focused on foundational knowledge related to glycemic control, while clinical assessments evaluated critical thinking, technical proficiency, and adaptability in real-time care settings.

Supervision and oversight: A continuous monitoring system led by head nurses and department-level teaching supervisors was established to ensure effective training implementation. Particular attention was given to high-risk personnel, time-sensitive activities, and key operational steps, thereby promoting comprehensive coverage and minimizing practice-related risks.

(2) Patient-managed insulin administration system

A structured self-management protocol for insulin administration was developed for inpatients with diabetes, addressing the key stages of assessment, storage, injection, education, and shift handover. The protocol was informed by prior AE data and piloted in departments with high diabetes case volume. Feedback from the pilot was used to refine the system prior to hospital-wide implementation. Nursing staff were organized into study groups to ensure thorough understanding of system components. Regular oversight was provided by departmental nursing managers and the hospital’s diabetes specialist team to ensure fidelity and consistency in implementation.

(3) Insulin pump management in non-endocrine departments

A dedicated insulin pump management system was developed for use in non-endocrine departments, incorporating best practices identified through literature review and clinical experience. The system comprised the following components:

Assessment and physician coordination: Nursing staff conducted blood glucose assessments and collaborated with physicians to determine the necessity and configuration of insulin pump therapy.

Training and operation: Nursing staff received formal instruction in pump use, including proper application, operational procedures, and troubleshooting for common malfunctions.

Handover protocols: Standardized shift-change procedures were implemented to verify pump placement, assess skin condition at the puncture site, confirm residual insulin volume, and check pump functionality.

Visual reminder system: Bedside signage was introduced for patients using insulin pumps, serving as a standardized alert to healthcare personnel.

Maintenance and oversight: Departmental nursing managers designated specific personnel to manage and inventory all insulin pumps, with routine maintenance and inspection procedures established.

Encouraging innovation: Nursing staff were encouraged to contribute creative solutions to challenges in insulin pump management, fostering continuous quality improvement in nursing practice.

(4) Strengthening nursing compliance with the check system

An internal audit conducted by the nursing management department revealed a 72.5 % correct implementation rate of the check system during insulin administration, indicating notable safety risks. In response, the protocol titled “Doctor’s Order Verification and Processing Process for Oral, Injection, Infusion, and External Application Drugs” was revised. In addition, two visual educational tools – “Correct Check Video” and “Medication Error Adverse Event Warning Video” – were developed to promote adherence, illustrate common points of failure, and reinforce error-prevention awareness among nursing staff.

(5) Promoting patient engagement in medication safety through health education

A series of educational interventions were designed to enhance patient involvement in safety management. Initiatives such as the “Clinical Nurse Innovation Awareness Workshop” and “New Ideas Learning Work Promotion Meeting” supported the development of patient-centered educational strategies. Two educational resources, the Rainbow Atlas Promotion Booklet and a Trifold Health Knowledge Pamphlet, were created, providing detailed guidance on insulin administration, adverse reactions, and preventive measures. To ensure accessibility, the materials were also made available in video format to accommodate patients with limited literacy or mobility.

Monthly “Immersive Workshops” were conducted to actively engage patients in safety education. These workshops utilized the “Medication Safety Participation Guideline” to promote dialogue and shared responsibility in insulin use, thereby fostering greater awareness and encouraging patients to take an active role in their care.

### Evaluation indicators

To evaluate the effectiveness of the risk mitigation strategies implemented in the intervention group beginning in January 2024, the following outcome indicators were assessed:

(1) Incidence of insulin type administration errors=(Number of insulin type errors/Total number of insulin administrations) × 100 %
(2) Incidence of insulin dose administration errors=(Number of insulin dose errors/Total number of insulin administrations) × 100 %
(3) Incidence of delayed insulin administration=(Number of delayed insulin administrations/Total number of insulin administrations) × 100 %

In addition to these error-based metrics, the resilience and safety behavior levels of nursing staff were evaluated both before and after participation in the training program, to assess changes in professional performance and response to patient safety risks.

### Research tools

(1) General data questionnaire

A department-developed general information questionnaire was used to collect demographic and clinical data from patients receiving subcutaneous insulin therapy. The tool facilitated standardized data acquisition across the study population.

(2) Resilience Scale for Health Care Workers

Resilience among medical staff was assessed using the Resilience Scale for Health Care Workers, developed by Zhu et al. [12]. This instrument comprises 18 items distributed across four dimensions: decision-making and coping, interpersonal connection, rational thinking, and flexible self-adaptation. Each item is rated on a five-point Likert scale, ranging from 1 (“completely disagree”) to 5 (“completely agree”). Higher total scores reflect greater psychological resilience.

The scale has demonstrated strong psychometric properties. The overall Cronbach’s α coefficient reported by the original authors was 0.904, indicating high internal consistency. Reliability coefficients for individual dimensions ranged from 0.610 to 0.785 based on test-retest analysis. In the present study, the total Cronbach’s α coefficient was 0.949, confirming the scale’s reliability within this sample.

(3) Nurse safety behavior Scale:

The Nurse Safety Behavior Scale, revised by Rong and Zhu in 2009 [13], was employed to evaluate safety-related behaviors among nursing staff. The instrument includes 12 items, each rated on a five-point Likert scale: 1=Never, 2=Rarely, 3=Sometimes, 4=Often, 5=Always. Total scores range from 12 to 60, with higher scores indicating stronger adherence to safety practices. The scale demonstrated excellent internal consistency, with a Cronbach’s α coefficient of 0.93, supporting its reliability and suitability for assessing nursing safety behavior in clinical settings.

### Statistical analysis

Data were double-entered using EpiData 3.0 to ensure data entry accuracy. Statistical analyses were conducted using SPSS version 26.0. Categorical variables are presented as frequencies and percentages, while continuous variables are expressed as mean ± standard deviation (SD). Group comparisons were performed using chi-square tests for categorical variables and independent-samples t-tests for continuous variables. Prior to conducting t-tests, data normality was assessed using the Shapiro–Wilk test, and homogeneity of variance was evaluated using Levene’s test. A two-tailed p-value of <0.05 was considered to indicate statistical significance. Cases with missing data were excluded during the data collection phase to maintain analytical integrity.

### Ethics approval and consent to participate

This study was conducted with approval from the Ethics Committee of Shanxi Bethune Hospital. This study was conducted in accordance with the declaration of Helsinki. Written informed consent was obtained from all participants.

### Consent for publication

Not applicable.

## Results

### Comparison of general characteristics between the two groups

A total of 123 patients were included in the study, with 58 assigned to the control group and 65 to the intervention group. All variables passed the normality test. Among all participants, 82 (66.7 %) were male and 41 (33.3 %) were female. The mean age was 50.76 ± 15.60 years in the control group and 55.34 ± 13.14 years in the intervention group. No statistically significant differences were observed between the two groups in terms of demographic or clinical baseline characteristics (p>0.05), indicating comparability (Table 3).

**Table 3:** Comparison of general data between the two groups.

Variables	Control group (n=58)	Intervention group (n=65)	Statistic	p-Value
Age	50.76 ± 15.60	55.34 ± 13.14	t=−1.767	0.080
Disease course, years	11.53 ± 8.64	12.22 ± 8.18	t=−0.433	0.666
Length of hospital stay	6.24 ± 2.19	5.98 ± 1.77	t=0.719	0.474
Insulin injection cost (yuan)	444.49 ± 227.01	321.56 ± 258.97	t=2.805	**0.006**
Highest blood glucose during hospitalization	20.34 ± 4.63	16.14 ± 3.64	t=5.630	<0.001
Lowest blood glucose during hospitalization	5.82 ± 1.20	5.78 ± 1.37	t=0.183	0.855
Number of blood glucose measurements during hospitalization (times)	37.09 ± 15.23	33.94 ± 13.49	t=1.215	0.227
Continuous glucose monitoring cost (in RMB)	810.00 ± 197.99	426.80 ± 270.49	t=1.778	0.136
Hospitalization cost (in RMB)	6,092.53 ± 1,942.00	6,299.43 ± 1,692.98	t=−0.631	0.529
Insulin types	1.79 ± 0.64	1.55 ± 0.73	t=1.920	0.057
Types of hypoglycemic drugs	1.85 ± 0.82	2.09 ± 0.95	t=−1.211	0.229
Hypoglycemic treatment with oral medications			χ^2^=0.165	0.684
No	18 (31.03)	18 (27.69)		
Yes	40 (68.97)	47 (72.31)		
Diabetes-related complications			χ^2^=0.399	0.528
No	38 (65.52)	39 (60.00)		
Yes	20 (34.48)	26 (40.00)		
Complications			χ^2^=0.994	0.803
Peripheral neuropathy	3 (15.00)	5 (19.23)		
Peripheral vascular lesions	11 (55.00)	16 (61.54)		
Diabetic nephropathy	4 (20.00)	4 (15.38)		
Ketoacidosis	2 (10.00)	1 (3.85)		
Insulin injection method			χ^2^=10.818	**0.004**
Insulin pump	18 (31.03)	23 (35.38)		
Insulin pen	8 (13.79)	23 (35.38)		
Both	32 (55.17)	19 (29.23)		
Monitor blood glucose dynamically?			χ^2^=0.390	0.532
No	56 (96.55)	60 (92.31)		
Yes	2 (3.45)	5 (7.69)		

The bold values represent the statistical results with statistical significance (p<0.05). Specifically, the bolded data indicate that the differences between the groups (or before/after intervention) are statistically significant, meaning the observed differences are not due to random chance but to the actual effect of the intervention implemented in this study.

### Comparison of AE incidence and blood glucose stability between the two groups

**Figure 2:**
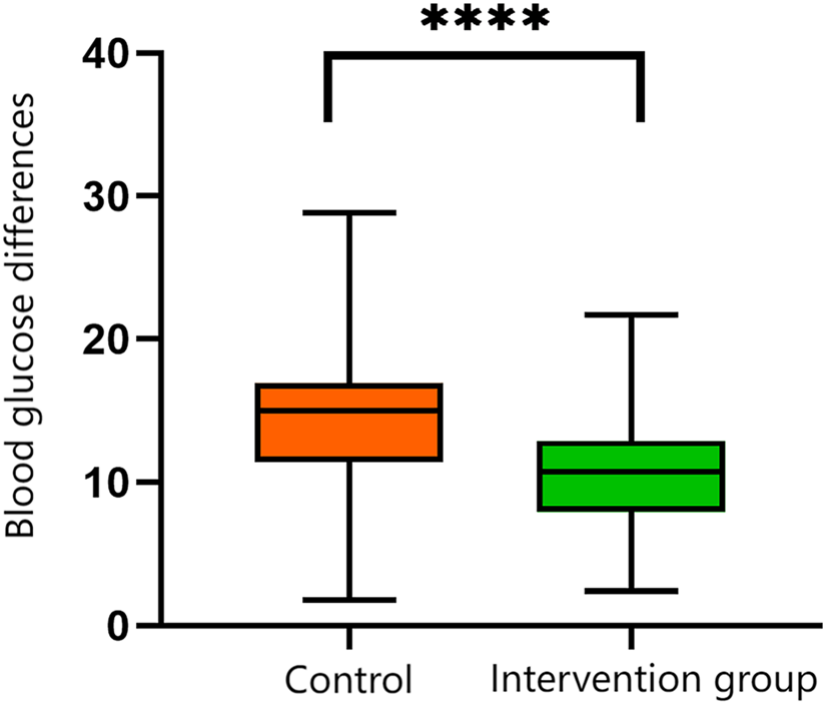
Comparison of blood glucose stability between the control and intervention groups. Asterisks indicate statistical significance. A greater number of asterisks corresponds to higher levels of significance, with **** denoting a statistically significant difference in blood glucose stability between the two groups.

The incidence rates of insulin type administration errors, dose errors, and delayed insulin injections were significantly lower in the intervention group compared to the control group (p<0.01) (Table 4). Furthermore, patients in the intervention group exhibited greater blood glucose stability than those in the control group, with the difference reaching statistical significance (Figure 2).

**Table 4:** Comparison of adverse events caused due to insulin medication errors between the two groups.

Variables	Control group (n=58)	Intervention group (n=65)	Statistic	p-Value
Insulin medication errors			χ^2^=22.575	**<0.001**
No	35 (60.34)	62 (95.38)		
Yes	23 (39.66)	3 (4.62)		
Insulin injection type error			χ^2^=10.573	**0.001**
No	47 (81.03)	64 (98.46)		
Yes	11 (18.97)	1 (1.54)		
Failure to inject insulin on time			χ^2^=5.101	**0.024**
No	50 (86.21)	64 (98.46)		
Yes	8 (13.79)	1 (1.54)		
Injection dose error			χ^2^=3.992	**0.046**
No	51 (87.93)	64 (98.46)		
Yes	7 (12.07)	1 (1.54)		

The bold values represent the statistical results with statistical significance (p<0.05). Specifically, the bolded data indicate that the differences between the groups (or before/after intervention) are statistically significant, meaning the observed differences are not due to random chance but to the actual effect of the intervention implemented in this study.

### Comparison of nursing staff resilience and safety behavior before and after RCA-based interventions

A total of 19 clinical nurses participated in the intervention. The mean length of service was 13.63 ± 9.72 years. Among the participants, seven nurses held primary professional titles, 11 held intermediate titles, and one held a deputy senior title. Following the implementation of RCA-based interventions, statistically significant improvements were observed in both resilience scores and safety behavior performance (p<0.01). Specifically, resilience scores increased by 24.23 %, and compliance with safety behavior improved by 10.9 %, indicating a positive impact of the RCA-informed measures (Table 5).

**Table 5:** Changes in resilience and safety behavior of nursing staff before and after training on improvement measures.

	Before training	After training	t	p-Value
Correct implementation of the check system	0.68 ± 0.48	0.95 ± 0.23	−2.041	0.056
Resilience	65.37 ± 6.71	81.21 ± 10.07	−6.540	**<0.001**
Decision-making and coping	21.68 ± 3.07	27.47 ± 3.61	−5.665	**<0.001**
Interpersonal connection	14.79 ± 3.01	18.37 ± 2.14	−4.539	**<0.001**
Rational thinking	14.63 ± 2.43	17.68 ± 2.67	−4.860	**<0.001**
Flexible self-adaptation	14.26 ± 2.08	17.37 ± 3.37	−4.331	**<0.001**
Safety behavior of nurses	52.30 ± 6.11	58.00 ± 2.66	−3.811	**0.001**

The bold values represent the statistical results with statistical significance (p<0.05). Specifically, the bolded data indicate that the differences between the groups (or before/after intervention) are statistically significant, meaning the observed differences are not due to random chance but to the actual effect of the intervention implemented in this study.

## Discussion

### Effect of integrated RCA on insulin-related AEs and blood glucose stability

Nursing AEs refer to unintended, unanticipated, or undesirable incidents occurring during the provision of nursing care [14]. These include events such as patient falls, medication errors, aspiration, burns, and other safety-related occurrences [15]. Reports indicate that more than 10 % of patients in developed countries experience medical harm, with higher rates observed in developing nations [16]. The incidence of AEs continues to rise, with an annual growth rate of approximately 0.63 % [17]. These events not only prolong hospital stays and increase economic burdens for patients but also impose significant psychological and occupational stress on healthcare personnel. Previous studies have shown that 50–60 % of healthcare workers have encountered AEs, which can result in lasting emotional and physical distress [18], 19].

Although nursing-related AEs cannot be entirely eliminated, many are preventable through structured management strategies. In China, AE management has been progressively refined, with increasing emphasis on the use of evidence-based tools to identify and address underlying causes. Among these tools, RCA has become a cornerstone in improving patient safety and clinical quality. In the present study, an integrated RCA approach was adopted to examine a series of insulin-related AEs. The methodology enabled the identification of systemic root causes and facilitated the development of standardized reporting and handover procedures, contributing to a substantial reduction in insulin-related medication errors.

The findings revealed a reduction in the incidence of insulin-related medication errors from 39.66 to 4.62 % following the intervention. Only one case of insulin type administration error was reported post-intervention, with marked decreases in the rates of delayed insulin administration and dosing inaccuracies. These outcomes underscore the effectiveness of the integrated RCA model in addressing complex, multifactorial causes of medication errors.

Comparative analysis with existing literature highlights a key contribution of this study. While previous RCA-based interventions have largely focused on isolated factors, such as educational training alone, this study employed a multi-faceted strategy combining workflow optimization, interdisciplinary collaboration, and active patient engagement. This comprehensive model yielded sustained improvements at a six-month follow-up, supporting the assertion that systemic RCA outperforms single-focus interventions in complex hospital environments. These findings align with previous research indicating that multi-dimensional quality improvement strategies are more effective in reducing nursing-related AEs [20], 21].

This study employed an integrated RCA approach to systematically examine a series of insulin-related AEs, identify root causes using evidence-based methodologies, and standardize reporting and handover protocols. These efforts led to a marked reduction in the incidence of insulin-related medication errors. As insulin administration has evolved from a specialized procedure primarily conducted in endocrinology departments to a routine responsibility for general nursing staff, the importance of accurate administration, structured handovers, and standardized protocols has become integral to effective medication management. Ongoing training in insulin preparation and administration remains a critical component of risk mitigation.

The implementation of this intervention was supported by clinical experts with extensive management experience, and the diabetes specialist team played a central role in promoting consistency and standardization across insulin management practices. Nursing personnel enhanced oversight of patient-owned insulin and strictly adhered to the “three checks and seven verifications” protocol during medication administration. These measures collectively contributed to a significant reduction in insulin-related medication errors. Moreover, patients in the intervention group demonstrated improved glycemic stability compared to those in the control group, underscoring the clinical effectiveness of standardized insulin management in enhancing patient outcomes.

Looking ahead, it is anticipated that hospitals will further strengthen medication management by integrating advanced information systems and decision-support technologies to enable intelligent and automated insulin management. Concurrently, expanded training for nursing staff and enhanced patient education programs will be critical in strengthening insulin self-management and reducing human-factor variability. These strategies are expected to further minimize the incidence of preventable insulin-related medication errors.

### Impact of integrated RCA on nursing staff resilience and safety behavior

A review of 15,037 AEs revealed that 37.3 % were recurrent, with both recurrent and non-recurrent events posing significant risks to patient safety, as reported in previous studies [22]. Nursing staff, as the primary point of contact in patient care, are central to ensuring clinical safety. Their resilience and adherence to safety behaviors are fundamental competencies that directly impact patient outcomes and contribute to the delivery of high-quality nursing care [12], 13]. Therefore, strengthening these attributes is essential in building a robust patient safety culture.

The occurrence of AEs can serve as a catalyst for professional growth, prompting reflection, reinforcing ethical standards, and improving clinical practice [23], 24]. Active learning and interprofessional collaboration have been shown to enhance patient safety outcomes [23]. In this study, the implementation of an integrated RCA approach resulted in a 24.23 % increase in nursing staff resilience scores and a 10.9 % improvement in safety behavior compliance. These improvements were likely facilitated by targeted training in insulin administration and reinforcement of standardized workflows and management protocols.

The RCA process enabled nursing staff to identify operational deficiencies, such as inconsistencies in insulin pump management, and apply evidence-based corrective actions. This structured, team-based approach likely contributed to the observed improvements in staff performance and safety outcomes. Compared with interventions using individual RCA, the integrated model adopted in this study fostered a proactive and collaborative safety culture. This model supported early identification and timely mitigation of potential AEs. In high-risk or emergency scenarios, nursing staff demonstrated improved responsiveness and adherence to standardized procedures, ultimately contributing to the provision of comprehensive, safe, and high-quality patient care.

### Study limitations

Several limitations of this study should be acknowledged. First, the single-center design, with the study conducted at a tertiary hospital in Shanxi Province, may limit the generalizability of the findings to other healthcare settings with different levels of resources, clinical workflows, or patient populations. Second, the use of convenience sampling (n=123) and a quasi-experimental design without randomization introduces the possibility of selection bias, which may affect the internal validity of the results. Third, the intervention period of six months may be insufficient to fully evaluate the long-term sustainability of improvements achieved through the integrated RCA intervention. Extended follow-up is needed to assess whether reductions in medication errors and improvements in nursing behavior are maintained over time. To enhance the robustness and external validity of future research, multi-center randomized controlled trials with longer follow-up periods (e.g., 12 months or more) are recommended. Furthermore, reliance on self-reported AE data in this study may introduce subjectivity or underreporting. The incorporation of objective data sources, such as electronic medication administration records, in future studies could improve data accuracy and reliability.

## Conclusions

This study underscores the effectiveness of integrated RCA in reducing insulin-related medication errors in inpatient settings. By systematically identifying underlying causes, such as workflow deficiencies and training inadequacies, and implementing targeted, multidisciplinary interventions, the approach led to a significant reduction in AEs, enhanced patient outcomes, and decreased the financial burden associated with preventable medical errors. The findings support the conclusion that integrated, system-level RCA strategies are more effective than isolated interventions in addressing complex safety challenges in clinical practice. These results align with evidence-based nursing practices and highlight the importance of sustained, structured risk management approaches to improve medication safety and overall care quality.
